# Simultaneous osseous metaplasia nodules of the submucosa and mesosalpinx after first trimester abortion: a case report

**DOI:** 10.1186/2047-783X-18-47

**Published:** 2013-11-19

**Authors:** Zhou Feng, Qin Jiale, Zhang Xiaofei, Guo Qingyun, Huang Lili

**Affiliations:** 1 Department of Pathology, Women’s Hospital, School of Medicine, Zhejiang University, Hangzhou, Zhejiang Province, China; 2 Department of Ultrasound, Women’s Hospital, School of Medicine, Zhejiang University, Hangzhou, Zhejiang Province, China; 3 Department of Obstetrics and Gynecology, Women’s Hospital, School of Medicine, Zhejiang University, Hangzhou, 1 Xueshi Road, Hangzhou, Zhejiang Province 310006, People’s Republic of China

**Keywords:** Osseous metaplasia, Nodules, Vaginal bleeding, Infertility

## Abstract

**Objectives:**

Here, we report a case of simultaneous osseous metaplasia nodules of the submucosa and mesosalpinx after a first trimester abortion.

**Case presentation:**

A 36-year-old woman presented to the Women’s Hospital, School of Medicine, Zhejiang University with vaginal bleeding and infertility resulting from osseous metaplasia nodules of the submucosa and mesosalpinx after a first trimester abortion. Diagnostic and operative hysteroscopy and laparoscope procedures were performed. The osseous metaplasia nodules disappeared after hysteroscopy and laparoscope interventions; 2 weeks postoperatively, the patient underwent a transvaginal ultrasound examination and the abnormal ultrasound appearance had resolved.

**Conclusions:**

Osseous metaplasia nodules in the submucosa and mesosalpinx can be a rare cause of vaginal bleeding and infertility. Autologous tissue, not persistent heterologous tissue, may be the true reason for metaplasia. Treatment is by ultrasound-guided curettage or by hysteroscopic and laparoscope removal.

## Background

Osseous metaplasia occurs in approximately 3 in every 10,000 women [1]. A history of abortion, either spontaneous or therapeutic, is the hallmark of this condition [2]. The etiology and pathogenesis are controversial. Retained fetal bones and inflammatory response following the abortion are thought to be the reasons for endometrial ossification [3].

Bony fragments in the uterus occur after second trimester termination of pregnancy [1-3]. Very rarely, bony fragments can form following first trimester loss [4]. The clinical presentation of osseous metaplasia can be pelvic pain, vaginal bleeding, menstrual irregularities, dyspareunia, and secondary infertility [2,3,5]. Osseous metaplasia is easily diagnosed by ultrasound examination, revealing an echogenic band, and diagnosis can subsequently be confirmed by hysteroscopy [1,2,6]. In this report, we present a rare case with osseous metaplasia involving the intrauterine submucosa and mesosalpinx after the first trimester loss.

## Case presentation

A 36-year-old woman was admitted to our hospital with a complaint of vaginal bleeding and infertility. Her medical history revealed she had regular menstrual cycles with no history of endocrine abnormalities. She had undergone four pregnancy terminations: one at 12 weeks’ gestation 12 years ago, one at 20 weeks’ gestation 9 years ago, one at 12 weeks’ gestation 6 years ago and one at 6 weeks’ gestation 2 years ago. The abortions were all electively terminated by dilatation and curettage. A pelvic examination revealed a normal size and normal adnexa. The results of laboratory investigations, which included a blood count, urine analysis, serum calcium, phosphorus, endocrine hormone levels and her partner’s spermiogram, were within normal limits.

**Figure 1:**
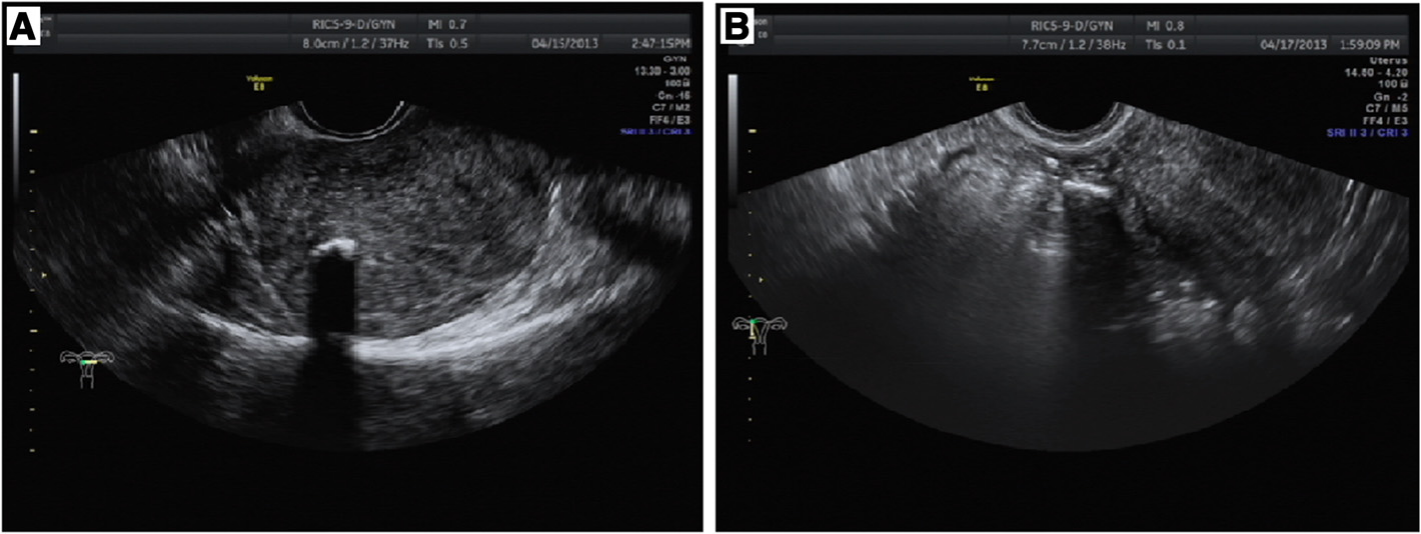
Two strong echogenic stripes with attenuation in the uterine submucosa (A) and mesosalpinx (B) in the transvaginal ultrasonography, respectively.

**Figure 2:**
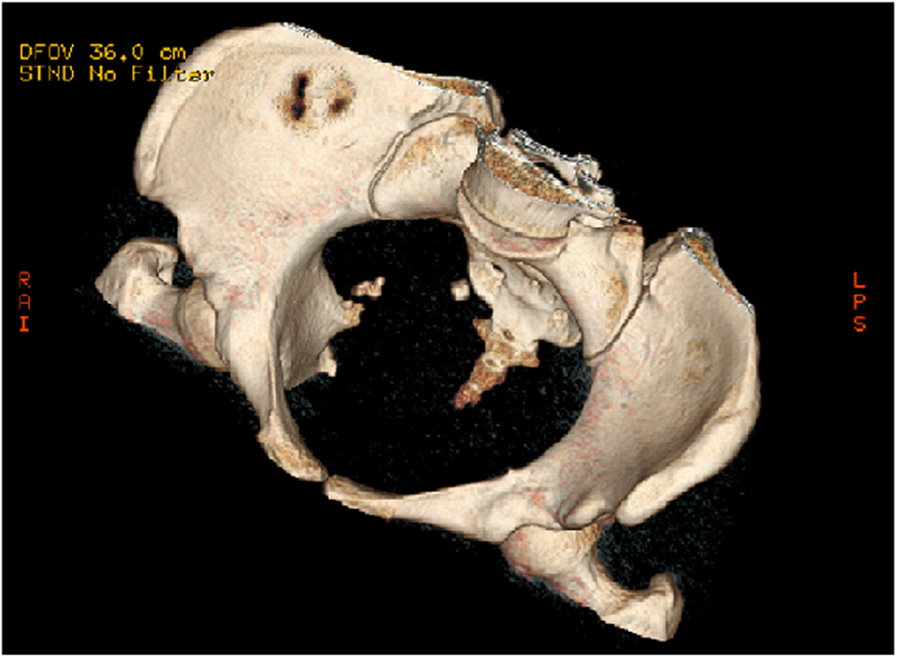
Two separate bony nodules were showed in the pelvic cavity by three-dimensional multislice computed tomography (MSCT-3D) examination.

**Figure 3:**
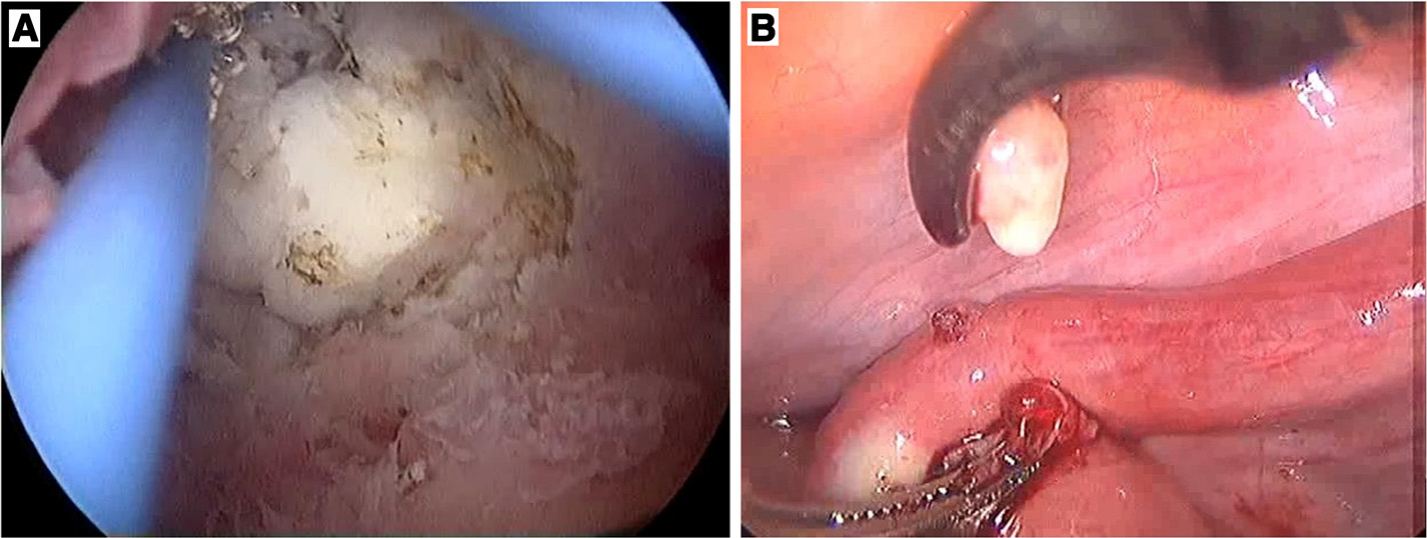
Two separate bony nodules in the submucosa (A) and mesosalpinx (B) were revealed by the diagnostic hysteroscopic and laparoscope examination.

**Figure 4:**
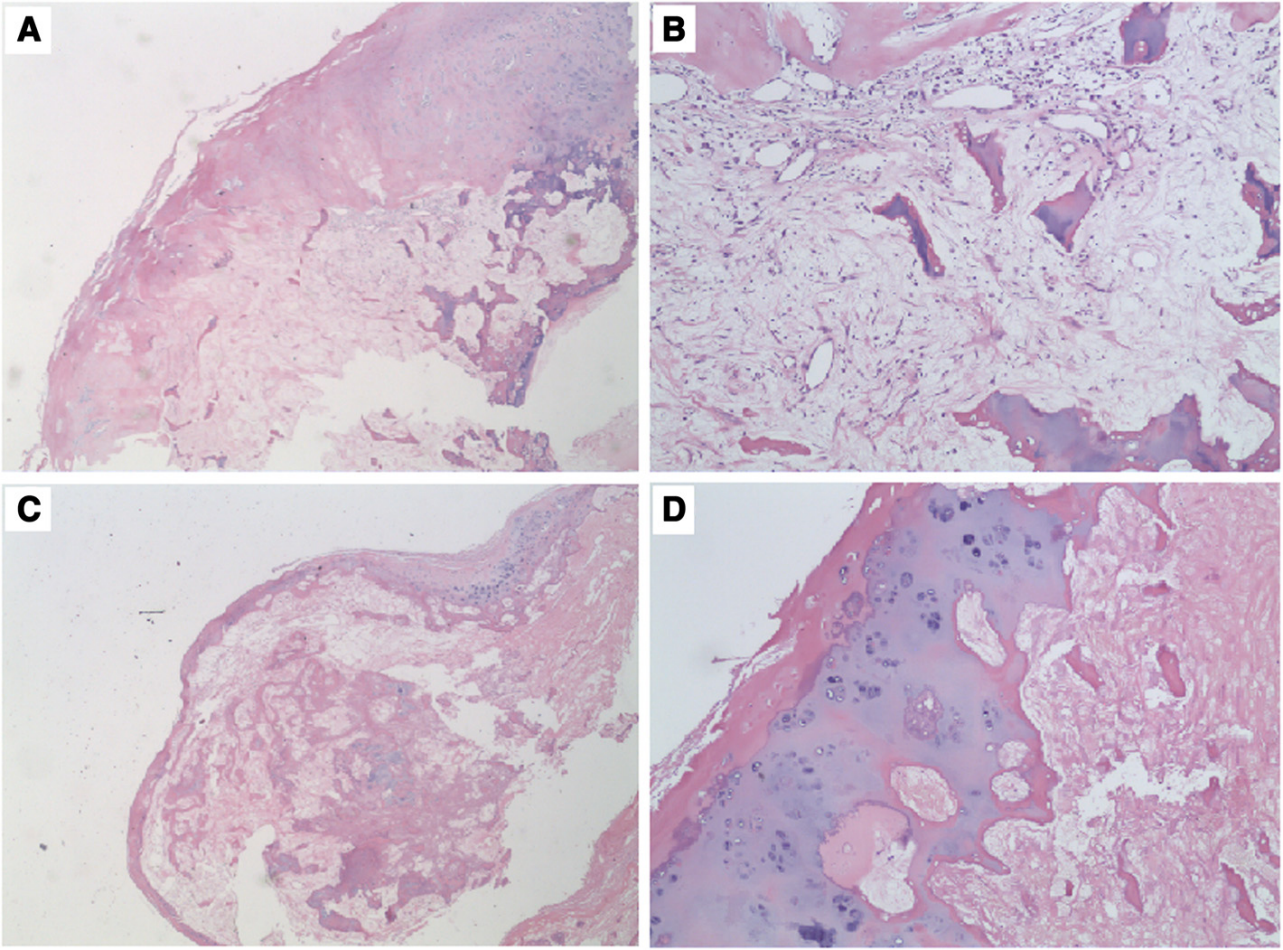
**The morphological characteristics of osseous metaplasia nodules of the submucosa (A,B) and mesosalpinx (C,D).** Sections were routinely stained with hematoxylin and eosin (HE). The low-power view shows a well-circumscribed nodule composed of peripheral lobules of mature hyaline cartilage (magnification × 25). The high-power view shows benign-appearing chondrocytes without atypia, and trabecular bone formation (magnification × 100).

During the tests, the transvaginal ultrasonography displayed two strong echogenic stripes with attenuation in the uterine submucosa and mesosalpinx (Figure 1). Two separate bony nodules were seen on three-dimensional multislice computed tomography (MSCT-3D) (Figure 2). Diagnostic hysteroscopic and laparoscope examinations performed in the operating room also revealed two separate bony nodules in the submucosa and mesosalpinx (Figure 3). A biopsy was obtained and the histology established the diagnosis of osseous metaplasia of the submucosa and mesosalpinx (Figure 4).

At 2 weeks postoperatively, the patient underwent a transvaginal ultrasound examination; the abnormal ultrasound appearance had resolved.

## Discussion

The ossification of the endometrium is a rare benign disease [6-8]. In the present case, the patient had a history of four terminations of pregnancy and osseous metaplasia nodules were found on transvaginal ultrasonography after the latest first trimester termination. First trimester abortions are an unusual cause of fetal bones, though this has been reported in a case previously [4]. The period of time between abortion and diagnosis of the disease can range from 8 weeks to 23 years [6,9]. In our case, the time interval was 2 years.

Ossification has also been reported in the cervix, the ovary, and the vagina [2,10-12]. To the best of our knowledge, this is the first reported case of osseous metaplasia nodules of both the submucosa and mesosalpinx. Persistent heterologous tissue, such as retained fetal bone, has been suggested as the etiology of osseous metaplasia of the endometrium in most reports [1-3]. However, it is unlikely that the endometrial bone is of fetal origin because there was no fetal tissue found in the biopsy material studied after the first trimester abortion, and the biopsy also showed minimal or no tissue reaction [13]. Thus, we predict that the osseous metaplasia may have a mullerian origin, arising in the myoendometrial transitional zone.

Studies have shown that chronic endometritis can stimulate the proliferation of mesenchymal cells that have inherent metaplasia properties and can differentiate into chondroblasts or osteoblasts [14]. Recently, Cayuela *et al*. [15] used DNA analysis to show that endometrial ossification is not of fetal origin, but derived from the patient’s own tissue. Additionally, our patient has undergone four curettage surgeries that may have resulted in chronic pelvic inflammation, causing the patient’s own tissue to differentiate into chondroblasts or osteoblasts. Therefore, persistent heterologous tissue may not be the cause of osseous metaplasia nodules of the mesosalpinx.

Interestingly, osseous metaplasia nodules, not multiple small and hard bony spicules, of the submucosa and mesosalpinx formed simultaneously in present case. Leiomyoma with osseous differentiation and mature teratoma can mimic osseous metaplasia nodules. However, our patient’s osseous metaplasia nodules contained neither mature smooth muscle areas nor epithelial components, which are the characteristics of uterine leiomyoma with osseous differentiation and mature teratoma, respectively.

## Conclusions

The diagnosis is suspected at ultrasound, MSCT-3D and confirmed by hysteroscopy, laparoscope and histopathologic examination. Treatment is by ultrasound guided curettage or by hysteroscopic and histopathologic removal. Autologous tissue, not persistent heterologous tissue, is resulting in a true metaplasia mostly.

## Consent

Written informed consent was obtained from the patient for publication of this case report and any accompanying images. A copy of the written consent is available for review by the Editor-in-Chief of this journal.

## Competing interests

The authors state they have no competing interests.

## Authors’ contributions

All authors made substantial contributions to conception and design, or acquisition of data, or analysis and interpretation of data; ZF and HL involved in drafting the manuscript and revising it critically for important intellectual content; HL gave final approval to the version to be published. All authors read and approved the final manuscript.

## Authors’ information

Zhou Feng and Qin Jiale are the joint first authors.
